# Rapid Learning of Earthquake Felt Area and Intensity Distribution with Real-time Search Engine Queries

**DOI:** 10.1038/s41598-020-62114-8

**Published:** 2020-03-25

**Authors:** Hengshu Zhu, Ying Sun, Wenjia Zhao, Fuzhen Zhuang, Baoshan Wang, Hui Xiong

**Affiliations:** 1 Baidu Inc., Beijing, 100085 China; 2 Key Lab of Intelligent Information Processing of Chinese Academy of Sciences (CAS), Institute of Computing Technology, CAS, Beijing, 100190 China; 3 University of Chinese Academy of Sciences, Beijing, 100049 China; 4 Institute of Geology, China Earthquake Administration, Beijing, 100029 China; 5 School of Earth and Space Sciences, University of Science and Technology of China, Hefei, 230026 China; 6 Rutgers, the State University of New Jersey, Newark, NJ 07102 USA

**Keywords:** Computational science, Natural hazards

## Abstract

Immediately after a destructive earthquake, the real-time seismological community has a major focus on rapidly estimating the felt area and the extent of ground shaking. This estimate provides critical guidance for government emergency response teams to conduct orderly rescue and recovery operations in the damaged areas. While considerable efforts have been made in this direction, it still remains a realistic challenge for gathering macro-seismic data in a timely, accurate and cost-effective manner. To this end, we introduce a new direction to improve the information acquisition through monitoring the real-time information-seeking behaviors in the search engine queries, which are submitted by tens of millions of users after earthquakes. Specifically, we provide a very efficient, robust and machine-learning-assisted method for mapping the user-reported ground shaking distribution through the large-scale analysis of real-time search queries from a dominant search engine in China. In our approach, each query is regarded as a “crowd sensor” with a certain weight of confidence to proactively report the shaking location and extent. By fitting the epicenters of earthquakes occurred in mainland China from 2014 to 2018 with well-designed machine learning models, we can efficiently learn the realistic weight of confidence for each search query and sketch the felt areas and intensity distributions for most of the earthquakes. Indeed, this approach paves the way for using real-time search engine queries to efficiently map earthquake felt area in the regions with a relatively large population of search engine users.

## Introduction

Earthquake is a major natural geological disaster, inflicting huge economic losses and casualties worldwide every year. Although the precise short-term earthquake predictions are still difficult to achieve, the impact of significant earthquakes can be largely mitigated through the rapid estimation of post-disaster information and quick response decisions^1^. As a result, the significance of real-time seismology has been substantially recognized, and considerable progress has been made in this area during the past decades^2–4^. Along this line, a major research focus of real-time seismological community is to efficiently estimate the felt area and extent of ground shaking, immediately after a significant earthquake occurs, which provides critical guidance for government emergency response teams to conduct orderly rescue and recovery operations in the damaged areas.

Traditionally, in a few regions with densely-deployed seismic observation stations, the ground motion parameters can be accurately detected^5–7^, and be used to automatically estimate the earthquake felt area and intensity distributions through their quantitative relationship^8–10^, such as the implementation of ShakeMap system^11^ and GRSmap^12^. However, the construction of a dense seismic observation network for earthquake early warning is not trivial, which is always accompanied with huge financial cost and unavoidable difficulties due to the constraints of field environment or local policies. Therefore, for most regions with limited observation stations, as the current situation in China, the earthquake felt area is mainly mapped based on the result of field investigation by seismologists, and assisted with inference methods like empirical attenuation relationship models^13,14^. However, this kind of approach often costs a couple of hours to days, sometimes even months, with heavy labor burden, and cannot cover a wide range of regions. For example, as reported by China Earthquake Administration (CEA) officially, it costed about four days for drawing the intensity map of ${M}_{S}6.5$ Ludian Earthquake (Yunnan, China, August 3, 2014)^15^.

Instead, researchers have made many efforts on finding cost-effective alternatives to estimate the earthquake felt area, such as the estimation based on remote sensing data from geographic information system (GIS)^16–18^ and GPS trajectories from mobile devices^19,20^. With the development of the Internet, it becomes possible to collect user-reported macro-seismic data in a more convenient manner. The online feedbacks can be regarded as a kind of “crowd sensors” and intuitively tend to correlate with the disaster distribution. For example, the “Did You Feel It?” (DYFI) system^21^ developed by United States Geological Survey (USGS) and the LastQuake app^22^ developed by European-Mediterranean Seismological Centre (EMSC) have been collecting information from people who felt earthquakes and creating maps that show what people experienced and the extent of expected damage. Moreover, tweets also have been shown as an effective way to reflect people’s feelings of earthquake shaking, which can help to facilitate the construction of earthquake early warning systems^23–28^. However, due to the reasons of the limited popularity (e.g., earthquake information websites and apps), the non-real-time mechanism of information sharing (e.g., Twitter-like social media), or the noisy information of alternative data, the efficiency, coverage and reliability of these approaches usually cannot be guaranteed.

**Figure 1:**
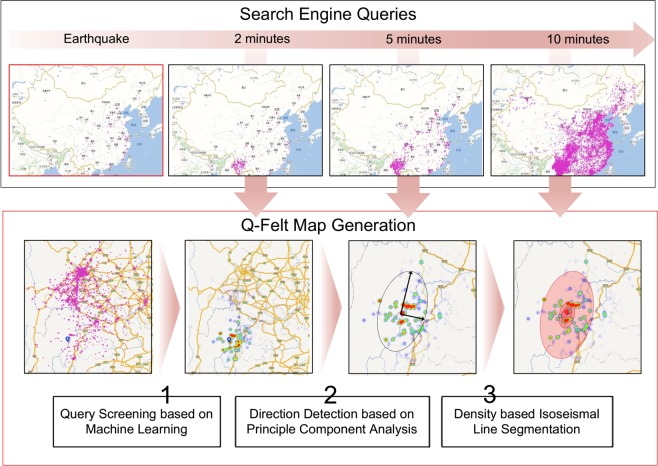
A schematic diagram of generating *Q-Felt Map*. By continuously monitoring the online search queries that contain the keyword “Earthquake” after an earthquake occurred, we can roughly obtain the geographical distribution of earthquake reports from users who felt the ground shaking and wanted to seek exact information online. To reduce the noisy information contained in original queries, and retain effective earthquake reports for drawing the *Q-Felt Map*, we first design a machine learning method to screen the original queries with weighting strategy. Then, we use the Principle Component Analysis (PCA) algorithm to estimate the direction of the semi-major axis of isoseismal lines, which are finally segmented by a density-based clustering algorithm. Indeed, the *Q-Felt Map* could be constantly revised with the update of new submitted queries.

To this end, here we introduce a new way for estimating the earthquake felt area and intensity distribution through monitoring the real-time information-seeking behaviors in the search engine queries^29,30^, which are submitted by tens of millions of users after earthquakes. Usually, when an earthquake occurs, people who felt the shaking will be urgent to know what exactly happened. In most cases, online search engine is the first and most convenient choice to obtain such information, compared with other information sources. Therefore, by monitoring the spatial-temporal distribution of real-time search queries that contain keyword “Earthquake”, we can estimate the felt area distribution in a timely manner. Specifically, we propose a very efficient, robust and machine-learning-assisted approach, namely *Query based Felt-area (Q-Felt) Map*, to sketch the user-reported ground shaking distribution, through the large-scale analysis of real-time search queries from a dominant search engine in China. In our approach, each query is regarded as a “crowd sensor” with certain weight of confidence to proactively report the shaking location and extent. Figure 1 shows a schematic diagram of our *Q-Felt Map*, which consists of three major components, namely *Query Screening based on Machine Learning*, *Direction Detection based on Principle Component Analysis (PCA)*, and *Density based Isoseismal Line Segmentation*. We validate our *Q-Felt Map* based on the large-scale search engine queries and historical earthquakes occurred in mainland China from 2014 to 2018. Experimental results clearly demonstrate that our approach can efficiently and realistically sketch the felt area maps for most of the earthquakes occurred in mainland China, by using real-time search queries after the earthquake. This approach paves the way for using real-time search engine queries to efficiently map earthquake felt area in the regions with a relatively large population of search engine users.

## Framework

**Figure 2:**
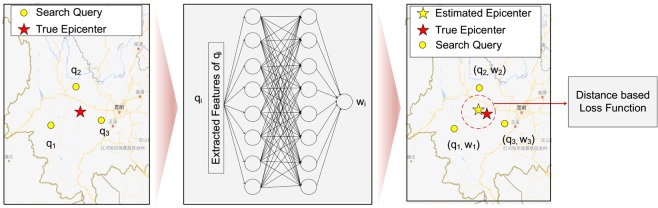
A schematic diagram of *Query Screening based on Machine Learning*. In our approach, every search query ${q}_{i}$ is associated with a weight of confidence ${w}_{i}$, which can be learned by a 3-layer deep neural network (DNN). Specifically, to build the loss function, we assume that if the weights are appropriately estimated, the weighted average (i.e., centroid) of the query locations can be used to estimate the epicenters of earthquakes.

**Figure 3:**
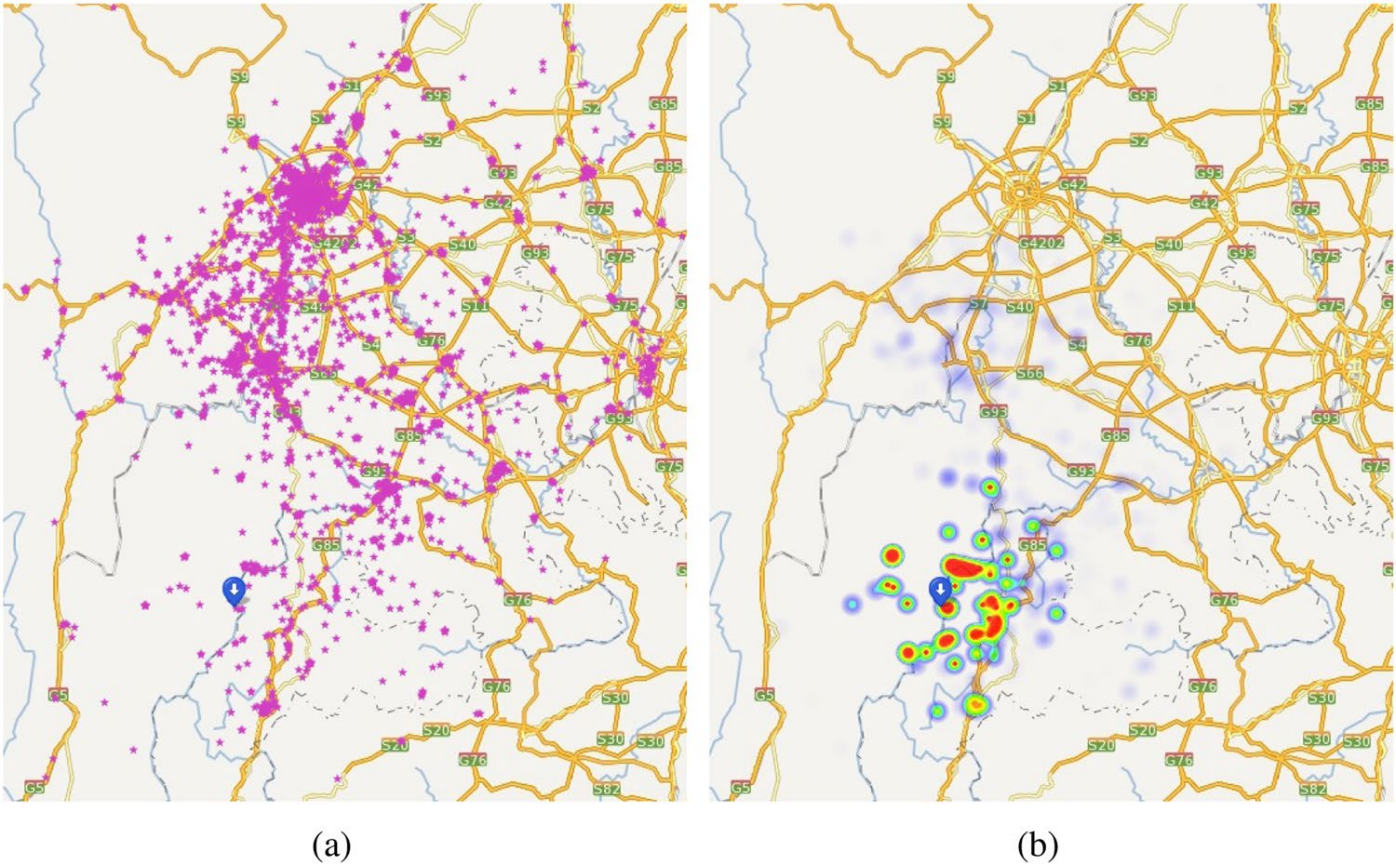
The distribution comparison between original search queries and screened search queries submitted within 5 minutes after ${M}_{S}5.0$ Yongshan earthquake (Yunnan, China, August 17, 2014). (**a**) The geographical distribution of original search queries, where each purple point denotes a query contains the keyword “Earthquake”, and the blue pin denotes the epicenter. (**b**) The heat map distribution of screened search queries, where each query is weighted by machine learning algorithm. All the maps were created by Baidu Map Open Platform JavaScript API v3.0 (http://lbsyun.baidu.com/index.php?title=jspopular3.0).

Intuitively, if we simply draw the distribution based on the density of search queries, the results will be overwhelmed by the regions that have large population of search engine users, such as the metropolis around. Meanwhile, not all the search queries are relevant to the occurred earthquake due to the diverse search intent. Therefore, it is necessary for us to find a way for screening the queries, i.e., filter out the noisy information contained in the massive query data. Accordingly, we regard each search query has a weight of confidence, which indicates the effectiveness of this query in terms of an earthquake sensor. Generally, the weight will be influenced by a number of factors, such as the submitted time, location, and surrounding population of query. For example, a query submitted right after the earthquake, which is from a location with relatively sparse population, is more properly to be an effective report. To estimate the weights of queries, we propose a machine learning approach, as shown in Fig. 2. Specifically, for each query ${q}_{i}$ submitted after an earthquake, we represent it as a $d$ dimensional feature vector ${{\bf{f}}}_{i}\in {{\mathbb{R}}}^{d}$, and introduce a machine learning model to learn a transformation function ${w}_{i}=g({{\bf{f}}}_{i};\beta ):{{\mathbb{R}}}^{d}\to {\mathbb{R}}$ that projects the feature vector to the weight of the query (i.e., ${w}_{i}$). In particular, we assume that if the weights are appropriately estimated, which means the parameter $\beta$ of function $g$ are well learned, the weighted average (i.e., centroid) of the query locations can be regarded as a proxy point that should be close to the epicenter of the earthquake. Based on this assumption, we can build a loss function for learning the coefficients, by minimizing the overall distance between estimated and real epicenters over historical earthquakes (see details in Method). As shown in Fig. 3, by screening the queries with their weights, the heat points of queries (i.e., the weight distribution of queries) move around the epicenter instead of the densely populated areas highlighted by original queries.

Traditionally, the isoseismal line is widely adopted for describing the felt area and extent of earthquake shaking. Therefore, in the *Q-Felt Map*, we also use isoseismal line as the indicator for sketching the felt areas of people-perceived ground shaking. Along this line, the first task is how to determine the directions of semi-major axis (or semi-minor axis) based on the screened queries. Here, we use the weighted PCA algorithm to project the locations of weighted queries onto new orthogonal coordinate system (see details in Method). After that, the obtained two eigenvectors can be used as the directional vectors, where the direction of eigenvector with larger value is for semi-major axis. Intuitively, the isoseismal line can be generalized by a series of concentric ellipses. Therefore, in the *Q-Felt Map*, we use the estimated epicenter as the origin of coordinate, and the eigenvalues are used for determining the flattening of ellipse.

**Figure 4:**
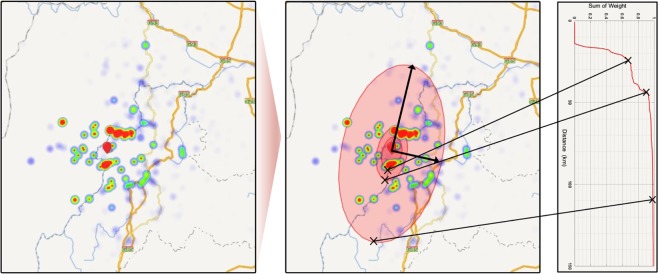
A schematic diagram of drawing isoseismal lines in *Q-Felt Map*. Generally, a *Q-Felt Map* can be represented by several concentric ellipses, the boundaries of which are segmented by isoseismal lines that indicate different extent of earthquake reports. With the direction of semi-major axis and flattening of ellipse learned by PCA from screened search queries, we can draw isoseismal lines by checking the change points in the distribution of accumulative weight sum of queries (shown in the right side). All the maps were created by Baidu Map Open Platform JavaScript API v3.0 (http://lbsyun.baidu.com/index.php?title=jspopular3.0).

Furthermore, we need to determine the value of semi-major axis and semi-minor axis length in isoseismal line, which is used to segment districts with different extent of ground shaking. To this end, here we design a density-based segmentation algorithm to draw the isoseismal (see details in Method). Figure 4 shows an example of drawing isoseismal line in our *Q-Felt Map*. Intuitively, the isoseismal lines are used to describe various degree of felt intensities. Although the districts segmented by isoseismal lines in the *Q-Felt Map* cannot be directly mapped into traditional intensity scales, such as the Modified Mercalli Intensity scale, the isoseismal lines can still be regarded as an alternative indicator for indexing the distribution and attenuation of people-perceived shaking intensity. Therefore, our *Q-Felt Map* provides a brand-new perspective on mapping the intensity distribution of earthquake.

## Results

**Figure 5:**
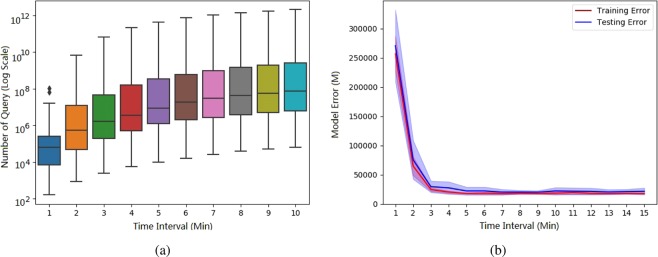
How many queries are sufficient for training our machine learning model? (**a**) The box plot of the amount of queries containing keyword “Earthquake” with respect to different time intervals after an earthquake occurred in our dataset. Indeed, the amount of queries in first 5 minutes increases sharply, and becomes stable afterwards. (**b**) The x-axis denotes the length of time window for accumulating search queries, and y-axis denotes the training/test error of our model with corresponding queries through a standard five-fold cross validation. Indeed, after 5 minutes, the errors become relatively stable and convergent, which indicates that using more queries will not significantly influence the learning results.

To validate the feasibility of our approach, we collected a dataset that contains the records of earthquakes occurred in mainland China from June 2014 to June 2018, from the China Earthquake Network Center (CENC), and conducted a standard five-fold cross validation on selected 554 earthquakes for training the machine leaning model (see details in Method). Actually, although the model can be constantly revised according to the update of queries, an open question is how many queries should be enough for learning the weights? To this end, we evaluated the performance of our machine learning model by selecting queries within different time intervals after earthquake as experimental data. From the results shown in Fig. 5, we can observe that, in our dataset, more than 1 million queries with keyword “Earthquake” on average were submitted within 3 minutes after each earthquake, the number of which is much larger than that of other alternative data in previous studies^21^. Meanwhile, we find that both training and testing errors become relatively stable and convergent, which indicates that using more queries will not significantly influence the learning results. This indicates that, for most cases, it could be enough for only using queries 5 minutes after the earthquake. After obtaining the optimal coefficients, we can compute the weight for each query.

Based on the above results, we explored to draw *Q-Felt Maps* for all the 58 earthquakes with magnitude $\ge {M}_{S}5.0$ in the dataset. In particular, to avoid the influence of noise, we filtered out the records where less than 10 non-duplicated search queries submitted within a 300 km radius of the epicenter and 10 minutes after the earthquake occurred. Consequently, 51 out of 58 earthquakes were retained for drawing the *Q-Felt Maps* by leveraging search queries only 5 minutes after the earthquake (see Supplementary Fig. S1–S4). For the filtered 7 records (see Supplementary Fig. S5), 6 earthquakes occurred in the sparsely populated areas of Tibet, where the population of search engine users is very limited. Interestingly, a special case is the ${M}_{S}6.4$ Linkou Earthquake (Heilongjiang, China, January 2, 2016), which occurred around the towns with large population but limited queries were submitted. Indeed, the focal depth of this earthquake is very deep (i.e., 580 km), thus no obvious ground shaking was felt by dwellers around.

**Figure 6:**
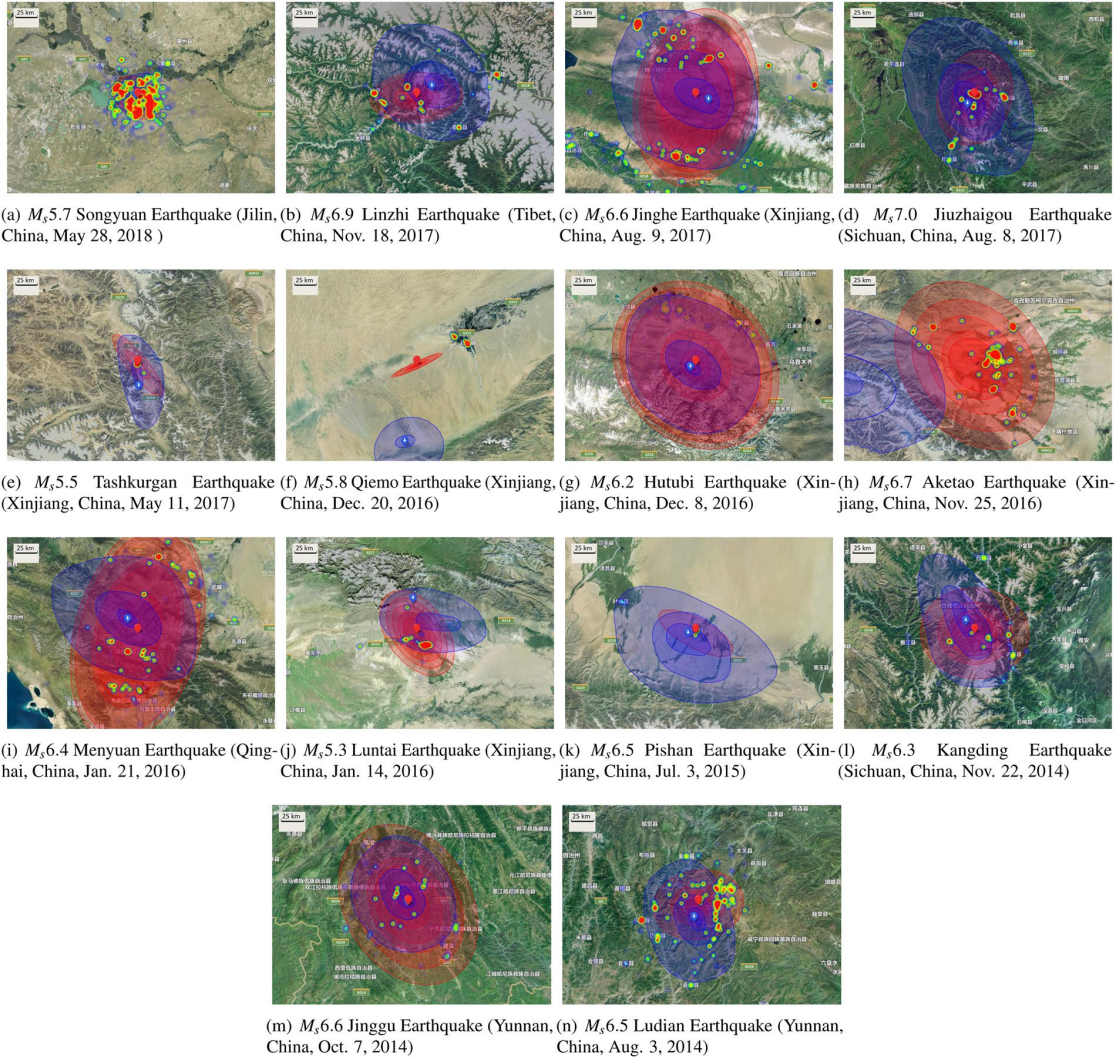
The comparison between *Q-Felt Maps* and official earthquake intensity maps. In each figure, the blue pin and area denote the epicenter and isoseismal area in official intensity map based on field investigation, respectively; the red pin and area denote the estimated epicenter and isoseismal area based on the online search queries within 5 minutes after the corresponding earthquake, respectively. The maximum length of the semi-major axis of ellipse is limited to 100 km. All the maps were created by Baidu Map Open Platform JavaScript API v3.0 (http://lbsyun.baidu.com/index.php?title=jspopular3.0).

In China, due to the limitation of earthquake emergency response mechanism, the CENC only reports official intensity maps, instead of felt maps, for parts of destructive earthquakes. Although the intensity map focuses more on the observed effects of the shaking (i.e, the extent and severity of damage to different kinds of structures or natural features), it still can be regarded as an alternative benchmark for validating the felt maps. Therefore, to further validate the effectiveness, we also compared the *Q-Felt Maps* of 14 earthquakes with their official intensity maps provided by China Earthquake Network Center, which were drawn through the field investigation of many seismologists, as shown in Fig. 6 (the results of *Q-Felt Maps* learned with queries 10 minutes after earthquakes are listed in Supplementary Fig. S6, which have similar distributions). We can observe that most of the earthquakes have relatively high similarity and region overlap for both maps, except for the ${M}_{S}5.8$ Qiemo Earthquake (Xinjiang, China, December 20, 2016) and ${M}_{S}6.7$ Aketao Earthquake (Xinjiang, China, Nov. 25, 2016), as shown in Fig. 6f,h, respectively. Therefore, the efficiency and robustness of our *Q-Felt Maps* have been validated.

## Discussion

Indeed, the population of online search engine users around the epicenter plays an important role in drawing *Q-Felt Map*, which directly affects the number of submitted search queries within the disaster regions. Intuitively, as shown in Fig. 6f,h, both the two earthquakes occurred in the sparsely populated areas, and relevant queries were submitted from towns that are located far away from the epicenter. In this case, the *Q-Felt Map* fails to highlight the exact disaster distribution. However, if the epicenter is near the densely populated areas, the situation will be totally different, such as the *Q-Felt Map* of ${M}_{S}5.5$ Tashkurgan Earthquake (Xinjiang, China, May 11, 2017) shown in Fig. 6e, which is drawn with only 161 queries. Fortunately, since all of the queries were submitted from the Tashkurgan Town, which is very close to the epicenter, the *Q-Felt Map* can still capture the queries and sketch the meizoseismal area.

Meanwhile, it is interesting to see that the *Q-Felt Maps* and official intensity maps of some earthquakes have large overlapped region but different directions of semi-major axis (e.g., as shown in Fig. 6b,c,i,n.) This phenomenon reveals another unique characteristic of our *Q-Felt Map* compared with traditional seismic intensity map. Specifically, since the official intensity maps are drawn based on the field investigation, the direction of semi-major axis is usually followed by the terrain of disaster regions, such as the fault trend. Differently, the *Q-Felt Map* is based on the “crowd sensors”, the shape of which reflects the distributions of user reported ground shaking. Therefore, the *Q-Felt Map* provides an alternative view-point on the impact of earthquakes.

In practice, another critical issue is about the computational efficiency of drawing *Q-Felt Maps*. Indeed, since the machine learning model for query screening can be pre-trained, the online process of drawing *Q-Felt Maps* is very efficient. Specifically, we evaluated the efficiency of drawing *Q-Felt Maps* for all the 58 earthquakes with magnitude $\ge {M}_{S}5.0$ and corresponding queries within 5 minutes in our dataset, on a typical personal computer (1.4GHz Dual-Core CPU and 4GB RAM). The average computation time is about $1.22$ seconds, which is very short compared with the time of collecting queries (i.e., 5 minutes).

In summary, we show that it is possible to exploit real-time online search queries for rapidly drawing the felt areas of earthquakes, even about 5 minutes after earthquake. Therefore, the proposed *Q-Felt Map* could be an effective supplement of the traditional intensity map, especially for earthquakes occurred in areas with a relatively large population of online search engine users.

## Methods

### Privacy

None of the queries in the database of this project can be associated with a particular individual. The database only retains information about the submission time and GPS coordinates of queries that contains the Chinese keyword “Earthquake”, instead of information about the identity of any user. Furthermore, any of original search logs are being processed and used in accordance with Baidu’s privacy policy (https://www.baidu.com/duty/yinsiquan.html).

### Data Description

In this study, two kinds of data were used, namely the earthquake records and the search engine query records.

- The earthquake records and corresponding intensity maps were provided by the China Earthquake Network Center (CENC). Each record contains the magnitude, the GPS coordinates of the epicenter, and the initial time of an earthquake occurred during June 1st, 2014 to June 1st, 2018. To avoid the noise for learning the weights of queries, we only retained the earthquakes with magnitude no less than ${M}_{S}5.0$, and parts of the earthquakes with magnitude no less than ${M}_{S}3.0$. In addition, we filtered out the records where less than 10 non-duplicated search queries submitted within a 300km radius of the epicenter and 10 minutes after the earthquake occurred. As a result, there were 554 earthquakes for our experiments (the details of all earthquake records can be found in the Supplementary Information).
- The search query records were provided by Baidu Inc. Each record only consists of the submission time and GPS coordinates of queries that contain the Chinese keyword “Earthquake”. In this study, we used the search queries submitted within 5 minutes after the earthquakes to validate the efficiency of our approach, while some experimental results by using search queries within 10 minutes can also be found in the Supplementary Information.

### Query Screening based on machine learning

For each query ${q}_{i}$ submitted after an earthquake, we first extracted a feature vector ${{\bf{f}}}_{i}\in {{\mathbb{R}}}^{d}$, where $d$ denotes the dimension of the feature vector. Then we used a regression model to learn a transformation function $g:{{\mathbb{R}}}^{d}\to {\mathbb{R}}$ that projects the feature vector to the weight of the query.

#### Feature extraction

The dimension of the feature vector might be different with respect to different length of time interval for learning query weight. In this study, we totally extracted 122 one-hot features for each query submitted within 5 minutes after an earthquake occurred. Specifically, given an earthquake $e$ occurred at ${t}_{e}$ with epicenter ${l}_{e}$, and a query ${q}_{i}$ submitted from location ${l}_{i}$ at ${t}_{i}$ ($0\,min\le ({t}_{i}-{t}_{e})\le 5\,min$), the extracted feature vector ${{\bf{f}}}_{{\bf{i}}}$ is the concatenation of three representation feature vectors with one-hot encoding, namely ${{\bf{f}}}_{i}^{(time)}$, ${{\bf{f}}}_{i}^{(loc)}$, and ${{\bf{f}}}_{i}^{(pop)}$.

- **The representation of query time**. We defined a one-hot feature vector ${{\bf{f}}}_{i}^{(time)}=({\phi }_{i1}^{(time)},...,{\phi }_{iN}^{(time)})$ to represent the submitted time *t*_*i*_ of query ${q}_{i}$, where ${\phi }_{ik}^{(time)}$ is a characteristic function that equals to 1 if and only if ${t}_{i}$ belongs to the day period ${D}_{k}$ and $({t}_{i}-{t}_{e})$ belongs to the time interval ${T}_{k}$, and equals to 0 otherwise. Specifically, we segmented the value of ${T}_{k}$ and ${D}_{k}$ into several discrete intervals, i.e., ${T}_{k}\in \{[0:00AM,6:00AM),[6:00AM,12:00PM),[12:00PM,18:00PM),[18:00PM,24:00PM)\}$, and ${D}_{k}\in \{[0\,min,1\,min),[1\,min,2\,min),[2\,min,3\,min),[3\,min,4\,min),[4\,min,5\,min]\}$. As a result, the dimension of ${{\bf{f}}}_{i}^{(time)}$ equals to 20.
- **The representation of query location**. We defined a one-hot feature vector ${{\bf{f}}}_{i}^{(loc)}=({\phi }_{i1}^{(loc)},\ldots ,{\phi }_{iN}^{(loc)})$ to represent the submitted location ${l}_{i}$ of query ${q}_{i}$, where ${\phi }_{ik}^{(loc)}$ is a characteristic function that equals to 1 if and only if the distance between ${l}_{i}$ and epicenter ${l}_{e}$ is within the range of ${L}_{k}$, and equals to 0 otherwise. Specifically, we segmented the value of ${L}_{k}$ into 51 discrete intervals, where the first 50 intervals is segmented by every 10 kilometers from the epicenter ${l}_{e}$, the last interval is for those beyond 500 kilometers.
- **The representation of population density**. We defined a one-hot feature vector ${{\bf{f}}}_{i}^{(pop)}=({\phi }_{i1}^{(pop)},\ldots ,{\phi }_{iN}^{(pop)})$ to represent the population of search engine users around the submitted location ${l}_{i}$ of query ${q}_{i}$, where ${\phi }_{ik}^{(pop)}$ is a characteristic function that equals to 1 if and only if the population of the region grid, where the query submitted from, is within the range of ${P}_{k}$, and equals to 0 otherwise. Specifically, we split the whole Chinese mainland with 10 km $\times$ 10 km grids, and accumulated the amount of daily search queries to indicate the population within. Specifically, we segmented the value of ${L}_{k}$ into 51 discrete intervals, where the first 50 intervals is segmented by every 10,000 queries, and the last interval is for those beyond 500,000 queries.

#### Model implementation

For each query ${q}_{i}$, we used a 3-layer deep neural network (DNN) to learn its weight ${w}_{i}$. There are two hidden layers in the network, where each layer has 8 hidden units and the third layer outputs the weight of the query. In our approach, we assume that the well-learned weights of queries could be used for estimating the epicenter of earthquakes. Therefore, we used the distance between the weighted average of queries locations and the epicenter as loss function. Specifically, to facilitate the computation, we first transformed all the GPS coordinates through the Mercator projection. Let ${Q}_{k}$ be the query set after earthquake ${e}_{k}$, the estimated GPS coordinate $({\widetilde{{\mathcal{X}}}}_{k},{\widetilde{{\mathcal{Y}}}}_{k})$ of the epicenter can be formulated as ${\widetilde{{\mathcal{X}}}}_{k}=\frac{{\sum }_{{q}_{i}\in {Q}_{k}}{w}_{i}\cdot {x}_{i}}{{\sum }_{{q}_{i}\in {Q}_{k}}{w}_{i}},{\widetilde{{\mathcal{Y}}}}_{k}=\frac{{\sum }_{{q}_{i}\in {Q}_{k}}{w}_{i}\cdot {y}_{i}}{{\sum }_{{q}_{i}\in {Q}_{k}}{w}_{i}},$ where $({x}_{i},{y}_{i})$ is the Mercator coordinate of query ${q}_{i}$. Therefore, given *N* earthquakes with official coordinates $({\mathcal{X}},{\mathcal{Y}})$ of epicenter as ground truth, we can train the DNN by minimizing the average squared Euclid distance, i.e., ${\beta }^{* }=\arg {\min }_{\beta }\frac{1}{2N}{\sum }_{k=1}^{N}\left({({\widetilde{{\mathcal{X}}}}_{k}-{{\mathcal{X}}}_{k})}^{2}+{({\widetilde{{\mathcal{Y}}}}_{k}-{{\mathcal{Y}}}_{k})}^{2}\right).$

### Axis detection based on principle component analysis

After getting the weight of queries after earthquake ${e}_{k}$, we have a set of weighted points ${S}_{k}=\{({w}_{i},{x}_{i},{y}_{i})| {q}_{i}\in {Q}_{k}\}$. We suppose the *Q-Felt Map* is centered on the estimated epicenter $({\widetilde{{\mathcal{X}}}}_{k},{\widetilde{{\mathcal{Y}}}}_{k})$ and the direction of semi-major/minor axis can be obtained by conducting the weighted Principle Component Analysis (PCA) on ${S}_{k}$. In our experiments, the weighted PCA was implemented by the Statistics and Machine Learning Toolbox of Matlab. From the results of weighted PCA, we can obtain two eigenvectors $({{\bf{v}}}_{1},{{\bf{v}}}_{2})$, which are the directional vectors of semi-major axis and semi-minor axis of the *Q-Felt Map*, respectively. Meanwhile, the flattening of *Q-Felt Map* is computed as $\frac{| {{\bf{v}}}_{1}| -| {{\bf{v}}}_{2}| }{| {{\bf{v}}}_{1}| }$.

### Density based isoseismal line segmentation

The basic idea is to constantly enlarge the boundary of an ellipse from the origin or the boundary of the last ellipse, with the learned flattening, until there exists a sharp change of the weight sum of queries located within this ellipse. The intuition is if the regions have similar extent of devastation, the increase of the weight sum should be stable. As a results, for every sharp change, we can draw a concentric ellipse. Specifically, to draw the isoseismal lines, we first generated a set of concentric ellipses $E=\{{\varepsilon }_{1},{\varepsilon }_{2}...,{\varepsilon }_{m}\}$ according to the learned directional vectors and flattening, where the length of the semi-major axis of ${\varepsilon }_{i}$ equals $i$ kilometers and $m$ is the maximum length. Then, we built an array $A=({A}_{1},{A}_{2},...,{A}_{m})$, where ${A}_{i}$ equals to the weight sum of queries located within the ellipse ${\varepsilon }_{i}$. Furthermore, we conducted the second-order derivative on $A$, and used the indexes of top $k$ ($k=10$ in our experiments) results with highest absolute values as the length of semi-major axis for drawing isoseismal lines. To avoid that two isoseismal lines are too close to each other, we defined that the distance between to the semi-major axes of two adjacent isoseismal lines should be larger than $n$ kilometers (i.e, $n=5$ in our experiments), and the difference of their weight sums should be larger than a threshold (i.e, 0.005 in our experiments).

### Limitation

In this paper, due to the data limitation, we did not discuss the correlation between the felt intensity with some recorded ground motion parameters, which is indeed an interesting direction for future research.

## Supplementary information

Supplementary Information 1.

Supplementary Information 2.

## Data Availability

The seismic data that support our findings were provided by China Earthquake Network Center (CENC). All the data used in the experiments are available upon request.

## Code availability

All codes of this project are available upon request.
