# Supplementary material for: Rapid Learning of Earthquake Felt Area and Intensity Distribution with Real-time Search Engine Queries
Source: Sci Rep. 2020 Mar 25;10:5437. doi: 10.1038/s41598-020-62114-8 (PMC7096409; doi:10.1038/s41598-020-62114-8)
Supplement: Supplementary file 1 — Supplementary Information 1. [file 41598_2020_62114_MOESM1_ESM.pdf]

Supplementary Information for Rapid Learning of Earthquake Felt Area and Intensity Distribution with Real-time Search Engine Queries

Hengshu Zhu[1,*], Ying Sun[1,2,3], Wenjia Zhao[4], Fuzhen Zhuang[2,3], Baoshan Wang[5], and Hui Xiong[6,*]

[1]Baidu Inc., Beijing, 100085, China
[2]Key Lab of Intelligent Information Processing of Chinese Academy of Sciences (CAS), Institute of Computing Technology, CAS, Beijing 100190, China
[3]University of Chinese Academy of Sciences, Beijing 100049, China
[4]Institute of Geology, China Earthquake Administration, Beijing, 100029, China
[5]School of Earth and Space Sciences, University of Science and Technology of China, Hefei, 230026, China
[6]Rutgers, the State University of New Jersey, Newark, NJ 07102, U.S.A

* The corresponding authors (zhuhengshu@gmail.com, xionghui@gmail.com)

This PDF file includes:

Supplementary Figure S1 to S6

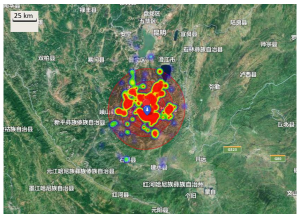

(a) M_s5.0 Yuxi Earthquake (Yunnan, China, Aug. 13, 2018)

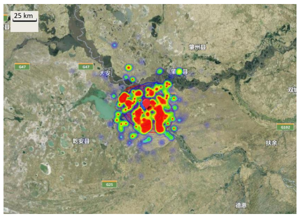

(b) M_s5.7 Songyuan Earthquake (Jilin, China, May 28, 2018)

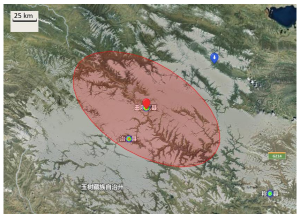

(c) M_s5.3 Yushu Earthquake (Qinghai, China, May 6, 2018)

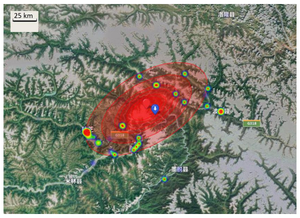

(d) M_s5.0 Linzhi Earthquake (Tibet, China, Dec. 20, 2017)

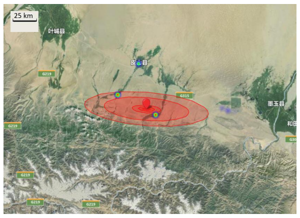

(e) M_s5.2 Yecheng Earthquake (Xinjiang, China, Dec. 7, 2017)

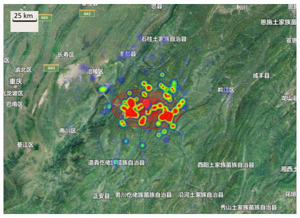

(f) M_s5.0 Wulong Earthquake (Chongqing, China, Nov 23, 2017)

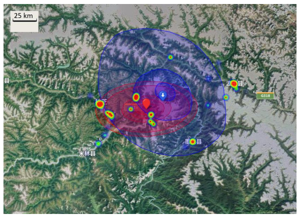

(g) M_s5.0 Linzhi Earthquake (Tibet, China, 8:31, Nov. 18, 2017)

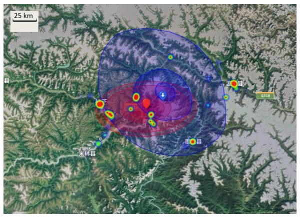

(h) M_s6.9 Linzhi Earthquake (Tibet, China, Nov. 18, 2017)

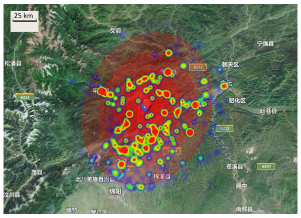

(i) M_s5.4 Qingchuan Earthquake (Sichuan, China, Sep. 30, 2017)

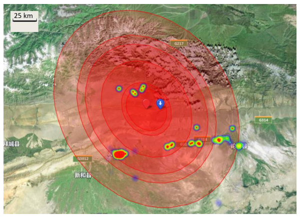

(j) M_s5.7 Akesu Earthquake (Xinjiang, China, Sep. 16, 2017)

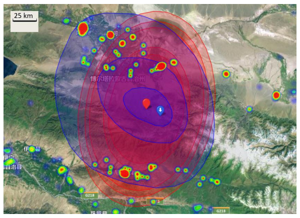

(k) M_s6.6 Jinghe Earthquake (Xinjiang, China, Aug. 9, 2017)

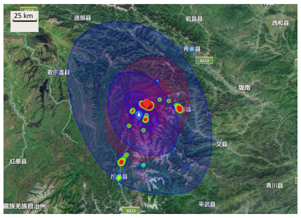

(l) M_s7.0 Jiuzhaigou Earthquake (Sichuan, China, Aug. 8, 2017)

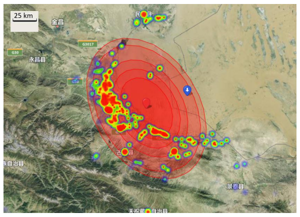

(m) M_s5.0 Alashan Left Banner Earthquake (Neimenggu, China, Jun. 3, 2017)

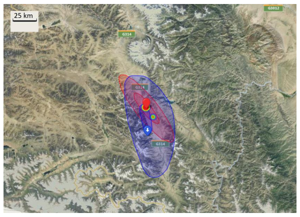

(n) M_s5.5 Tashkurgan Earthquake (Xinjiang, China, May 11, 2017)

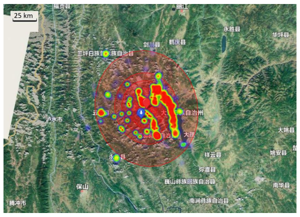

(o) M_s5.1 Yangbi Earthquake (Yunnan, China, Mar. 27, 2017)

Figure S1. The *Q-Felt Maps* of the earthquakes with magnitude $\geqslant M_s 5.0$ occurred in 2018 and 2017. In each figure, the blue pin and area denote the epicenter and isoseismal area in official intensity map based on field investigation, respectively; the red pin and area denote the estimated epicenter and isoseismal area based on the online search queries within 5 minutes after the corresponding earthquake, respectively. The maximum length of the semi-major axis of ellipse is limited to 100km. All the maps were created by Baidu Map Open Platform JavaScript API v3.0 (http://lbsyun.baidu.com/index.php?title=jspopular3.0).

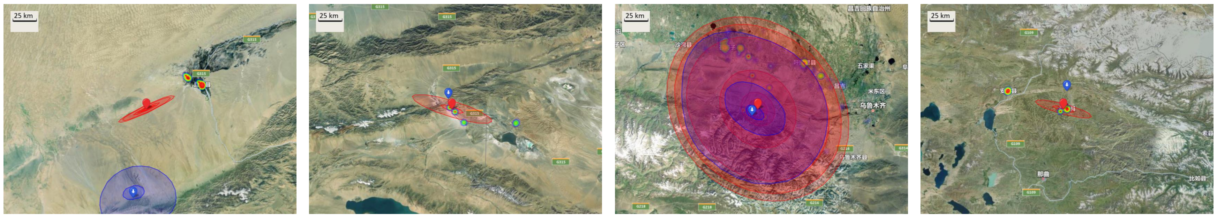

(a) M_s5.8 Qiemo Earthquake (Xinjiang, China, Dec. 20, 2016)

(b) M_s5.0 Ruoqiang Earthquake (Xinjiang, China, Dec. 14, 2016)

(c) M_s6.2 Hutubi Earthquake (Xinjiang, China, Dec. 8, 2016)

(d) M_s5.1 Nierong Earthquake (Tibet, China, Dec. 5, 2016)

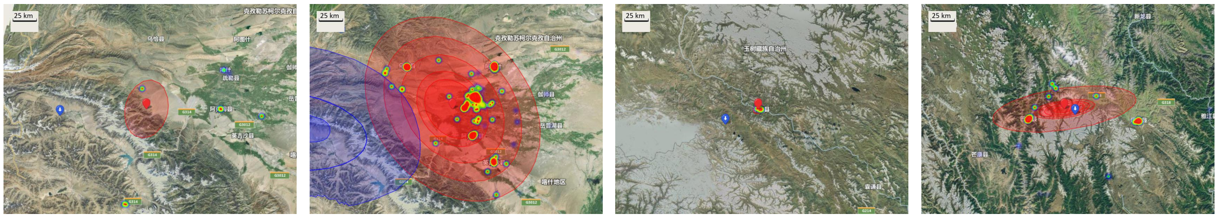

(e) M_s5.0 Aketao Earthquake (Xinjiang, China, Nov. 26, 2016)

(f) M_s6.7 Aketao Earthquake (Xinjiang, China, Nov. 25, 2016)

(g) M_s6.2 Yushu Earthquake (Qinghai, China, Oct. 17, 2016)

(h) M_s5.1 Ganzi Earthquake (Sichuan, China, Sep. 23, 2016)

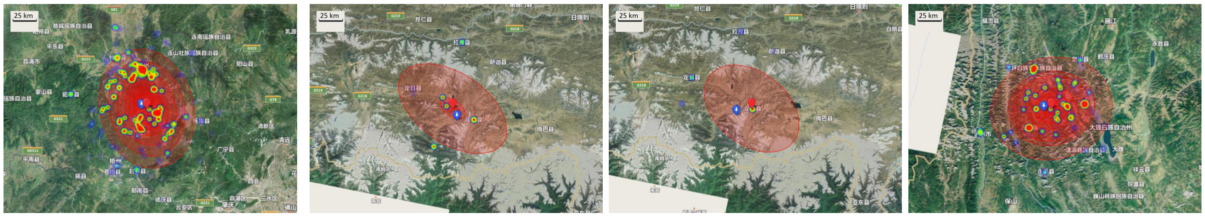

(i) M_s5.4 Cangwu Earthquake (Guangxi, China, July. 31, 2016)

(j) M_s5.3 Dingri Earthquake (Tibet, China, May. 22, 20161)

(k) M_s5.3 Dingjie Earthquake (Tibet, China, May. 22, 2016)

(l) M_s5.0 Yunlong Earthquake (Yunnan, China, May. 18, 2016)

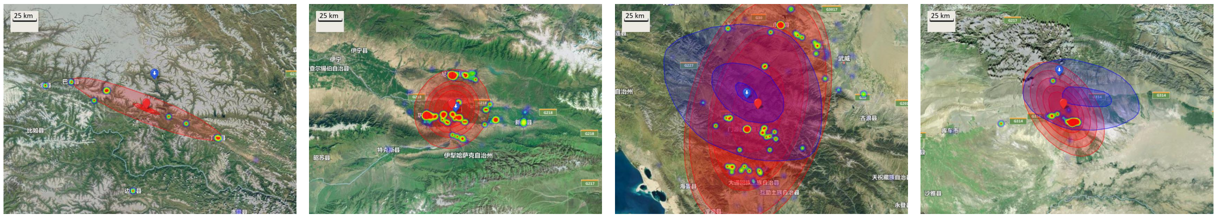

(m) M_s5.5 Dingqing Earthquake (Tibet, China, May. 11, 2016)

(n) M_s5.0 Yili Earthquake (Xinjiang, China, Feb. 11, 2016)

(o) M_s6.4 Menyuan Earthquake (Qinghai, China, Jan. 21, 2016)

(p) M_s5.3 Luntai Earthquake (Xinjiang, China, Jan. 14, 2016)

Figure S2. The *Q-Felt Maps* of earthquakes occurred in 2016. In each figure, the blue pin and area denote the epicenter and isoseismal area in official intensity map based on field investigation, respectively; the red pin and area denote the estimated epicenter and isoseismal area based on the online search queries within 5 minutes after the corresponding earthquake, respectively. The maximum length of the semi-major axis of ellipse is limited to 100km. All the maps were created by Baidu Map Open Platform JavaScript API v3.0 (http://lbsyun.baidu.com/index.php?title=jspopular3.0).

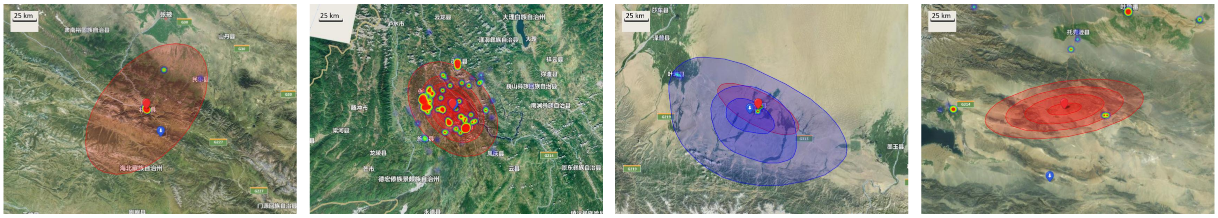

(a) M_s5.2 Qilian Earthquake (Qinghai, China, Nov. 23, 2015)
(b) M_s5.1 Changning Earthquake (Yunnan, China, Oct. 30, 2015)
(c) M_s6.5 Pishan Earthquake (Xinjiang, China, Jul. 3, 2015)
(d) M_s5.4 Toksun Earthquake (Xinjiang, China, Jun. 25, 2015)

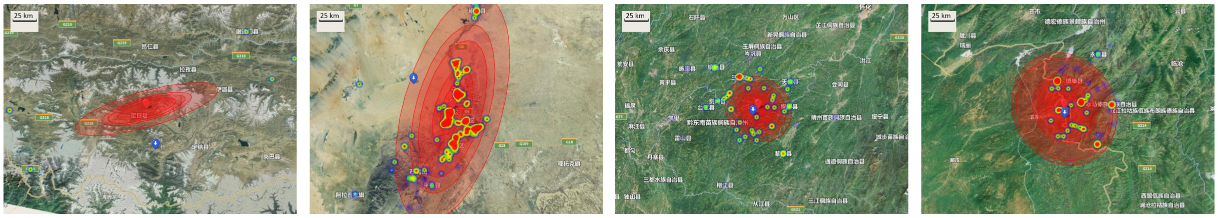

(e) M_s5.9 Dingri Earthquake (Tibet, China, Apr. 25, 2015)
(f) M_s5.8 Alashan Left Banner Earthquake (Inner Mongolia, China, Apr. 15, 2015)
(g) M_s5.5 Jianhe Earthquake (Guizhou, China, Mar. 30, 2015)
(h) M_s5.5 Cangyuan Earthquake (Yunnan, China, Mar. 1, 2015)

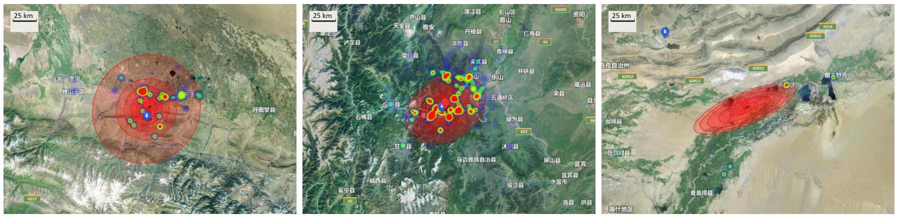

(i) M_s5.0 Shawan Earthquake (Xinjiang, China, Feb. 22, 2015)
(j) M_s5.0 Jinkouhe Earthquake (Sichuan, China, Jan. 14, 2015)
(k) M_s5.0 Atushi Earthquake (Xinjiang, China, Jan. 10, 2015)

Figure S3. The *Q-Felt Maps* of earthquakes occurred in 2015. In each figure, the blue pin and area denote the epicenter and isoseismal area in official intensity map based on field investigation, respectively; the red pin and area denote the estimated epicenter and isoseismal area based on the online search queries within 5 minutes after the corresponding earthquake, respectively. The maximum length of the semi-major axis of ellipse is limited to 100km. All the maps were created by Baidu Map Open Platform JavaScript API v3.0 (http://lbsyun.baidu.com/index.php?title=jspopular3.0).

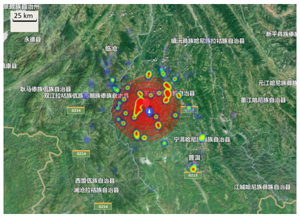

(a) M_s5.9 Jinggu Earthquake (Yunnan, China, 18:20, Dec. 6, 2014)

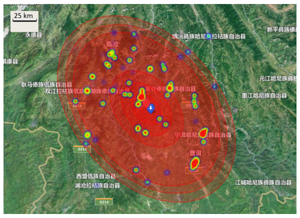

(b) M_s5.8 Jinggu Earthquake (Yunnan, China, 2:43, Dec. 6, 2014)

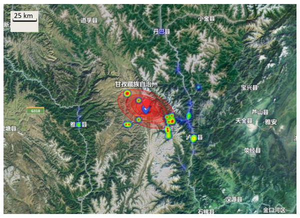

(c) M_s5.8 Kangding Earthquake (Sichuan, China, Nov. 25, 2014)

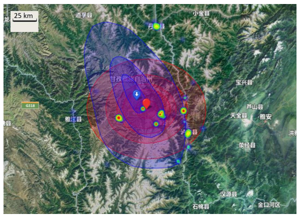

(d) M_s6.3 Kangding Earthquake (Sichuan, China, Nov. 22, 2014)

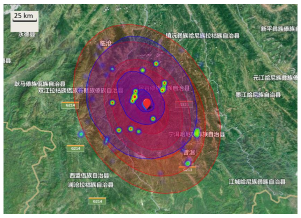

(e) M_s6.6 Jinggu Earthquake (Yunnan, China, Oct. 7, 2014)

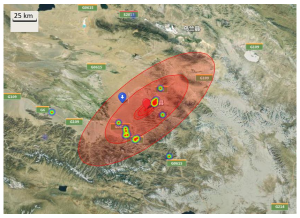

(f) M_s5.1 Kangding Earthquake (Qinghai, China, Oct. 2, 2014)

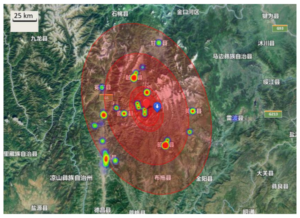

(g) M_s5.0 Yuexi Earthquake (Sichuan, China, Oct. 1, 2014)

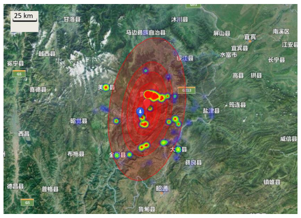

(h) M_s5.0 Yongshan Earthquake (Yunnan, China, Aug. 17, 2014)

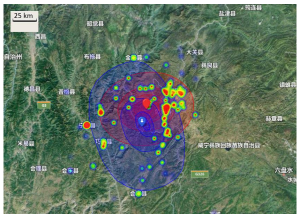

(i) M_s6.5 Ludian Earthquake (Yunnan, China, Aug. 3, 2014)

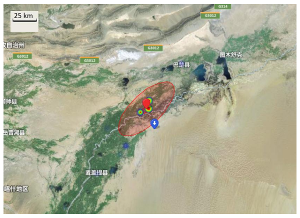

(j) M_s5.1 Maigaiti Earthquake (Xinjiang, China, Jul. 9, 2014)

Figure S4. The *Q-Felt Maps* of earthquakes occurred in 2014. In each figure, the blue pin and area denote the epicenter and isoseismal area in official intensity map based on field investigation, respectively; the red pin and area denote the estimated epicenter and isoseismal area based on the online search queries within 5 minutes after the corresponding earthquake, respectively. The maximum length of the semi-major axis of ellipse is limited to 100km. All the maps were created by Baidu Map Open Platform JavaScript API v3.0 (http://lbsyun.baidu.com/index.php?title=jspopular3.0).

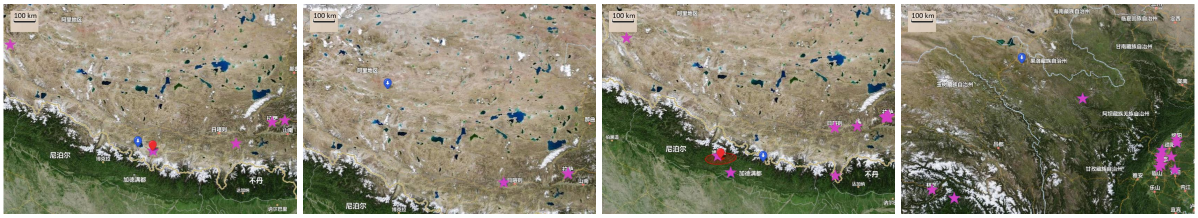

(a) $M_s5.0$ Jilong Earthquake (Tibet, China, Aug. 3, 2014)
(b) $M_s5.2$ Gaize Earthquake (Tibet, China, Feb. 2, 2015)
(c) $M_s5.3$ Nielamu Earthquake (Tibet, China, Apr. 26, 2015)
(d) $M_s5.2$ Maduo Earthquake (Qinghai, China, Oct. 12, 2015)

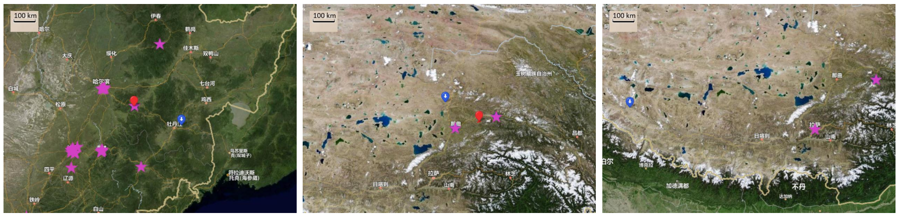

(e) $M_s6.4$ Linkou Earthquake (Heilongjiang, China, Jan. 2, 2016)
(f) $M_s5.3$ Anduo Earthquake (Tibet, China, Jan. 14, 2016)
(g) $M_s5.0$ Zhongba Earthquake (Tibet, China, Feb. 1, 2017)

Figure S5. The records with magnitude $\geq M_s5.0$, where less than 10 non-duplicated search queries submitted within a 300km radius of the epicenter and 10 minutes after the earthquake occurred. In each figure, the blue pin denotes the epicenter, and the pink stars denote relevant queries. All the maps were created by Baidu Map Open Platform JavaScript API v3.0 (http://lbsyun.baidu.com/index.php?title=jspopular3.0).

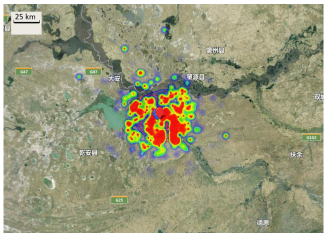

(a) M_s5.7 Songyuan Earthquake (Jilin, China, May 28, 2018)

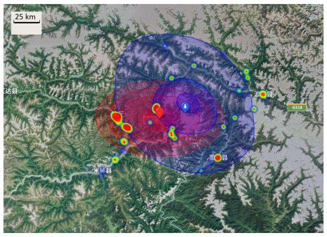

(b) M_s6.9 Linzhi Earthquake (Tibet, China, Nov. 18, 2017)

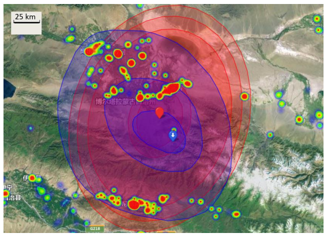

(c) M_s6.6 Jinghe Earthquake (Xinjiang, China, Aug. 9, 2017)

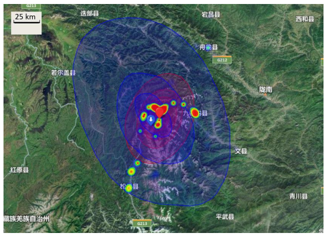

(d) M_s7.0 Jiuzhaigou Earthquake (Sichuan, China, Aug. 8, 2017)

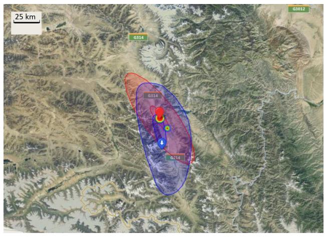

(e) M_s5.5 Tashkurgan Earthquake (Xinjiang, China, May 11, 2017)

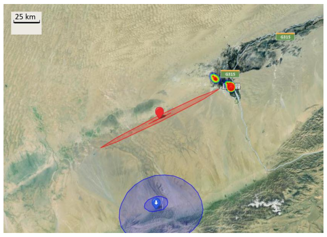

(f) M_s5.8 Qiemo Earthquake (Xinjiang, China, Dec. 20, 2016)

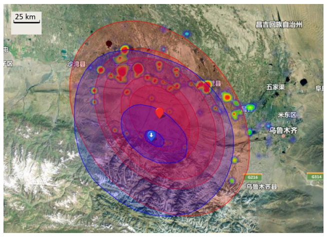

(g) M_s6.2 Hutubi Earthquake (Xinjiang, China, Dec. 8, 2016)

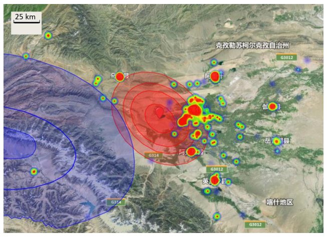

(h) M_s6.7 Aketao Earthquake (Xinjiang, China, Nov. 25, 2016)

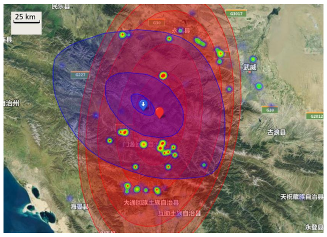

(i) M_s6.4 Menyuan Earthquake (Qinghai, China, Jan. 21, 2016)

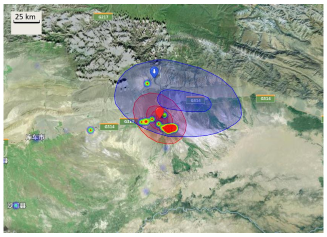

(j) M_s5.3 Luntai Earthquake (Xinjiang, China, Jan. 14, 2016)

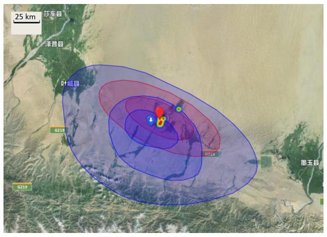

(k) M_s6.5 Pishan Earthquake (Xinjiang, China, Jul. 3, 2015)

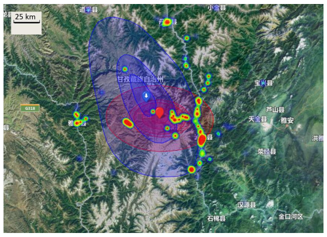

(l) M_s6.3 Kangding Earthquake (Sichuan, China, Nov. 22, 2014)

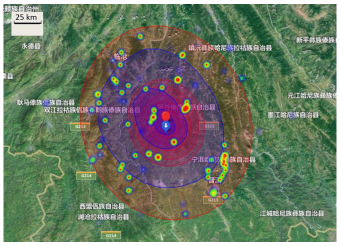

(m) M_s6.6 Jinggu Earthquake (Yunnan, China, Oct. 7, 2014)

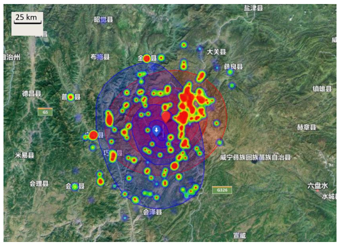

(n) M_s6.5 Ludian Earthquake (Yunnan, China, Aug. 3, 2014)

Figure S6. The comparison between *Q-Felt Maps* and official earthquake intensity maps with 10 minutes queries. In each figure, the blue pin and area denote the epicenter and isoseismal area in official intensity map based on field investigation, respectively; the red pin and area denote the estimated epicenter and isoseismal area based on the online search queries within 10 minutes after the corresponding earthquake, respectively. The maximum length of the semi-major axis of ellipse is limited to 100km. All the maps were created by Baidu Map Open Platform JavaScript API v3.0 (http://lbsyun.baidu.com/index.php?title=jspopular3.0).
